# Multi-Temporal Shoreline Monitoring and Analysis in Bangkok Bay, Thailand, Using Remote Sensing and GIS Techniques

**DOI:** 10.3390/jimaging12010021

**Published:** 2026-01-01

**Authors:** Yan Wang, Adisorn Sirikham, Jessada Konpang, Chunguang Li

**Affiliations:** 1 Faculty of Engineering, Rajamangala University of Technology Krungthep, Bangkok 10120, Thailand; 669041810054@mail.rmutk.ac.th (Y.W.); jessada.k@mail.rmutk.ac.th (J.K.); 2 School of Information Engineering, Jiangsu College of Finance and Accounting, Lianyungang 222061, China; lichunguang@jscfa.edu.cn

**Keywords:** remote sensing imagery, image processing, coastline changes, coastal engineering and environmental engineering

## Abstract

Drastic alterations have been observed in the coastline of Bangkok Bay, Thailand, over the past three decades. Understanding how coastlines change plays a key role in developing strategies for coastal protection and sustainable resource utilization. This study investigates the temporal and spatial changes in the Bangkok Bay coastline, Thailand, using remote sensing and GIS techniques from 1989 to 2024. The historical rate of coastline change for a typical segment was analyzed using the EPR method, and the underlying causes of these changes were discussed. Finally, the variation trend of the total shoreline length and the characteristics of erosion and sedimentation for a typical shoreline in Bangkok Bay, Thailand, over the past 35 years were obtained. An overall increase in coastline length was observed in Bangkok Bay, Thailand, over the 35-year period from 1989 to 2024, with a net gain from 507.23 km to 571.38 km. The rate of growth has transitioned from rapid to slow, with the most significant changes occurring during the period 1989–1994. Additionally, the average and maximum erosion rates for the typical shoreline segment were notably high during 1989–1994, with values of −21.61 m/a and −55.49 m/a, respectively. The maximum sedimentation rate along the coastline was relatively high from 2014 to 2024, reaching 10.57 m/a. Overall, the entire coastline of the Samut Sakhon–Bangkok–Samut Prakan Provinces underwent net erosion from 1989 to 2024, driven by a confluence of natural and anthropogenic factors.

## 1. Introduction

Conventionally, shoreline refers to the intersection of land and water surface at a selected tidal elevation, demarcating the marine–terrestrial boundary [1]. Coastlines are subject to irregular erosion and deposition due to human activities (e.g., construction of coastal infrastructure), natural disasters (e.g., tsunamis), natural factors (e.g., tides, waves, river flow, storms, geological structures) as well as morphological, climatic, or geological variables [2,3]. For tropical coastal zones like Bangkok Bay, monsoonal precipitation, seasonal variations in river runoff, and frequent extreme weather events further exacerbate coastline dynamics. In addition, sea-level rise has become an increasingly prominent driver of coastal erosion in this region, threatening coastal ecosystems and human settlements. These changes not only affect the migration rate of the coastline but also alter its direction of movement [4]. Shoreline dynamics have a direct bearing on the management of coastal zones and the planning of regional development [5]. Coastline change calculation is a fundamental technique for estimating coastal erosion and accretion, and for analyzing coastal morphological dynamics [6].

Traditional field survey methods for coastline extraction are time-consuming, labor-intensive, and costly, and they often lack long time series data, which hinders large-scale and multi-period surveys [7]. On the contrary, remote sensing technology provides the macroscopic, efficient, high precision, and cost-effective method to study the changes in coasts and has a specific edge in delineating coastlines [8]. Advanced maps have been generated through the development of remote sensing (RS) and geographic information systems (GISs), which have been exploited on the trending dynamic coastline [9]. Multi-temporal shoreline monitoring is widely used because a single year of data cannot show the full effects of tides, monsoon seasons, sediment movement, and human activities on coastal change. Long-term satellite records, such as those from Landsat, allow coastlines to be observed consistently over several decades. When these datasets are analyzed in a GIS environment, they help identify shoreline positions at different times and reveal clear patterns of erosion and accretion. Tools like the Digital Shoreline Analysis System (DSAS) further support this work by calculating standard shoreline change rates, including the End Point Rate (EPR), which is commonly used in long-term shoreline studies. The EPR approach, combined with satellite remote sensing, provides an effective method for assessing shoreline position changes at different times with high accuracy and reliability [10,11].

At present, based on the aforementioned remote sensing, GIS and DSAS and other technical methods, numerous studies have been commissioned on shoreline with significant attention being given on estuaries, harbors, islands, and other portions. This research is geared towards retrieving shorelines along different areas in a bid to check the erosion-accretion processes and identify the dynamics and the cause of the shoreline movement. Niang was able to estimate coastal change rates and spatial patterns of shoreline change along Saudi Arabia Yanbu coast with the help of multi-temporal satellite imagery, GIS, and the DSAS [12,13]. Nassar et al. investigated land change at the North Sinai of Egypt department in the form of multi-temporal satellite imaging in combination with the DSAS technique [14]. In the article by Thang et al., the authors have used the DSAS technique in delineating the shoreline change in the Kien Giang coastline in Vietnam [15]. Furthermore, using multi-temporal land satellite and GIS, Guneroglu assessed the change in the location of the coastline and land use in the northeast seaboard in Turkey to the Black Sea coast [16]. Mukhopadhyay et al. applied time-series Landsat data along with the EPR method to study shoreline changes and predict future shoreline positions at the Puri Coast, Bay of Bengal, India [17]. Salauddin et al. utilized multi-temporal satellite imagery combined with the DSAS technique to model shoreline dynamics of a coastal island in Bangladesh [18]. Thomas et al. have described a statistical analysis of the shoreline migration through the Tamil Nadu coast, which lies in the north of India, based on satellite remote sensing images over a long time period and employs the DSAS methodology [19]. Anastasiou and Sylaios assessed shoreline changes in the Nestos River Delta using satellite images and the LITPACK morphodynamic model [20]. Bui and Pham analyzed shoreline variations and erosion patterns in the Ca Mau Cape region of the Mekong Delta using multi-temporal satellite images and hydrodynamic modeling techniques [21]. Such a mechanism was explained by Pham et al. by interpreting the hydrodynamic model and remote sensing data to clarify the mechanism of the erosion zones formation along the Mekong Delta coast, Vietnam [22]. Based on an automated method, which entailed combining multi-temporal satellite images with GIS and the DSAS extension, Kermani et al. compared the dynamics of the functioning shoreline in the sandy coastal region of Jijelian, eastern Algeria [23].

However, minimal literature has been directed to the Gulf of Thailand. Bangkok Bay, located in the northern part of the Gulf of Thailand, is a typical tropical coastal zone with important ecological and socio-economic value. It is adjacent to the capital Bangkok and several economically developed provinces, where coastal resources support local fisheries, tourism, and port industries. Meanwhile, the bay is highly sensitive to natural and human disturbances: the intensive coastal construction (e.g., port expansion, reclamation), rapid mangrove degradation, and accelerating sea-level rise due to climate change have posed severe threats to coastal stability. However, the lack of systematic long-term monitoring of coastline changes in this key region has become a bottleneck for effective coastal zone management. For instance, Chowdhury and Tripathi investigated coastal erosion and accretion in southern Thailand’s Pak Phanang region over a 30-year period (1973–2003), using time-series topographic maps and Landsat satellite images [24]. Chusrinuan et al. undertook a comparison of shoreline evolution in southeastern Thailand’s Songkhla Province between 1975 and 2006 by analyzing aerial photographs and Landsat images integrated within GIS [25]. Additional literature addresses topics such as shoreline erosion due to sea-level rise, mangrove degradation, and the current state and methods of slope stabilization and erosion control [26,27,28]. There is a lack of systematic analysis of the Bangkok Bay area, especially regarding the long-term sequence and multi-temporal evolution characteristics of the coastline in this region.

In view of this, this study identifies a scarcity of research on Bangkok Bay, particularly regarding the long-term temporal and spatial variations in the coastline. The geographical scope of this investigation encompasses the coastal zones of Phetchaburi, Samut Songkhram, Samut Sakhon, Bangkok, Samut Prakan, Chachoengsao, Chon Buri, and Rayong. To examine the spatiotemporal dynamics of shoreline length, erosion, and accretion in Bangkok Bay, coastline information for five time points (1989, 1994, 2004, 2014, and 2024) was derived from Landsat TM/OLI medium-resolution remote sensing images. We employed the EPR method within the DSAS to assess the change rate of a representative shoreline segment over a 35-year period and further investigated the causative factors behind its transformation to support decision-makers with the data for integrating coastal zone management (ICZM).

## 2. Materials and Methods

### 2.1. Overview of the Study Area

The Gulf of Thailand, which was historically designated the Gulf of Siam, is positioned on the southern flank of the Indochina Peninsula in Southeast Asia. It is the southern gulf of the Kingdom of Thailand, bounded by the Indochina Peninsula to the north and the Malay Peninsula to the south. The gulf’s opening to the south exhibits an inverted “U” shape, extending approximately from Cape Cà Mau in Vietnam to Kota Baru in Malaysia. Geographically, it spans from 99°10′ E to 105°00′ E longitude and from 6°00′ N to 13°30′ N latitude. The gulf is approximately 720 km in length from north to south, with an east–west width ranging from 480 to 560 km, covering a total area of about 250,000 square kilometers. The Gulf of Thailand originated from tectonic processes, including crustal movement and faulting, during the Tertiary period (65 million to 2.6 million years ago). The Indochina Peninsula, where the gulf is located, predominantly experiences a tropical monsoon climate, characterized by the prevailing southwest monsoon during the summer months and a higher annual precipitation [29].

Recent studies have further validated the role of DSAS and GIS/RS in monitoring coastal dynamics. For instance, Fariz and Martuti analyzed shoreline change impacts on coastal communities in Indonesia using Landsat and DSAS, recommending spatial zoning measures for risk mitigation [30]. Similarly, Ambarwati and Siswanto assessed shoreline stability along Merah Putih Beach to inform protective intervention strategies [31]. These studies illustrate the growing precision and utility of modern tools in coastal zone management.

The integration of RS and GIS into coastal design projects has become increasingly common. For example, Cabezas-Rabadán et al. used remote monitoring to evaluate engineering interventions along Spain’s Valencian Coast [32]. Similarly, Pujianiki and Simpangan applied Sentinel-2 imagery and DSAS to analyze shoreline responses before and after harbor development in Bali [33]. These applications demonstrate the value of geospatial tools for adaptive infrastructure design and post-project assessment.

Shoreline monitoring should be considered not only alone as an environmental issue but also seen as an engineering design challenge. Accurate shoreline is important to the planning, laying, and maintenance of coastal defense structures. Shoreline position and rates of change directly influence the design of seawalls, revetment, groynes and beach nourishment works as well as forming resilience strategies within Integrated Coastal Zone Management (ICZM) structures.

Bangkok, the capital city of Thailand, is located within the northern Gulf of Thailand. The maritime region south of the metropolitan area is designated as “Bangkok Bay.” This bay serves as the estuary for several rivers in Thailand, including the Chao Phraya, Mampa Gong, Taqin, and Mekelon Rivers. The coastline along the Gulf features diverse shoreline types, such as aquaculture ponds, ports, mangrove forests, and river estuaries, making it a region of significant coastal use and change in Thailand.

**Figure 1:**
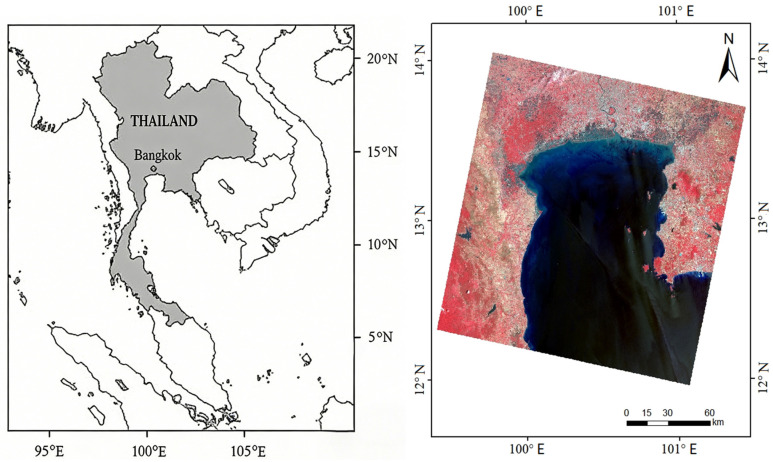
Geographical location of the investigated area.

Geographically, this research covers the Bangkok Bay coastline, encompassing the eight provinces of Phetchaburi, Samut Songkhram, Samut Sakhon, Bangkok, Samut Prakan, Chachoengsao, Chon Buri, and Rayong (Figure 1).

### 2.2. Data Source and Processing

**Figure 2:**
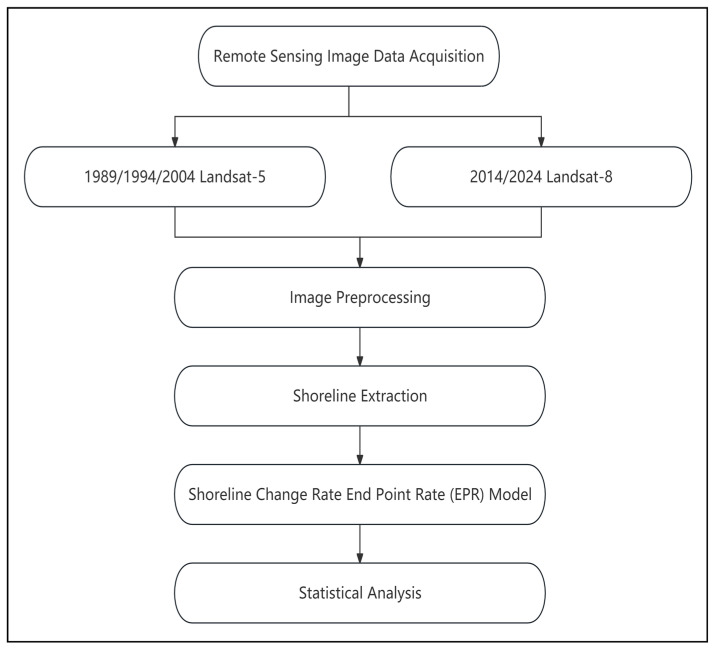
Flowchart of the overall methodology applied in this study.

Multi-temporal satellite imageries of Landsat 5 TM (manufactured by Hughes Aircraft Company, Los Angeles, California, USA) and Landsat 8 OLI (manufactured by Ball Aerospace & Technologies Corporation, Boulder, Colorado, USA) sensors (Table 1) have been utilized for the study area in 1989, 1994, 2004, 2014, and 2024, with a spatial resolution of 30 m and an acquisition interval of 5–10 years. To highlight the water-land boundary lines, a pseudo-color synthesis method was adopted. Specifically, the near-infrared, red, and green spectral bands were, respectively, assigned the colors red (R), green (G), and blue (B), and a pseudo-color synthesis was performed. Additionally, appropriate linear stretching was applied to enhance the visibility of the water-land boundaries. The Bangkok Bay shoreline change analysis study has followed the methodology workflow shown in Figure 2.

### 2.3. Image Preprocessing

To address the issues of spatial and radiometric consistency among multi-temporal satellite images and ensure the accuracy of coastline extraction and subsequent change analysis, this study implemented the following precise matching preprocessing steps for all five temporal phases of satellite imagery (1989, 1994, 2004, 2014, and 2024). All images were first unified to the WGS 1984 geographic coordinate system (EPSG: 4326) and subsequently projected to the WGS 1984 UTM Zone 47N projected coordinate system (EPSG: 32647) for spatial standardization. For Landsat 5 TM without official Level-2 products, radiometric calibration and FLAASH atmospheric correction (the Tropical atmospheric model and the hybrid Urban-Maritime aerosol model) were performed on Level-1T data to obtain surface reflectance, supporting MNDWI calculation and coastline extraction. For Landsat 8 OLI data, official Level-2 surface reflectance products were directly adopted, as these products have undergone preprocessing (including radiometric calibration and atmospheric correction) by the United States Geological Survey (USGS), Reston, Virginia, USA. Given the consistent 30 m spatial resolution of the core bands between Landsat 5 TM and Landsat 8 OLI sensors, cross-resolution resampling was deemed unnecessary. The above preprocessing procedures ensure the spatial and radiometric consistency of the multi-temporal dataset.

### 2.4. Coastline Extraction Method

In this study, the Modified Normalized Difference Water Index (MNDWI) was adopted for coastline extraction. The MNDWI is specifically designed for land-water separation, with its calculation based on the green band and short-wave infrared (SWIR) band of remote sensing images, as shown in Equation (1):

(1) $$\mathrm{MNDWI}=\frac{\mathrm{Green}-\mathrm{SWIR}}{\mathrm{Green}+\mathrm{SWIR}}$$

where

Green = Band 2 (Landsat 5 TM) or Band 3 (Landsat 8 OLI);

SWIR = Band 5 (Landsat 5 TM) or Band 6 (Landsat 8 OLI).

The MNDWI was calculated using ENVI 5.6 software to realize land-water separation, thereby obtaining preliminary coastline extraction results. Subsequently, in ArcMap 10.8.1 software, interactive visual interpretation and correction were performed on the preliminary coastlines of five historical periods (1989, 1994, 2004, 2014, and 2024) to generate standardized vector coastline data.

### 2.5. Calculation Method of Coastline Change

The End Point Rate (EPR) is a method that captures the net change characteristics of coastlines. Its primary advantage lies in its simplicity of calculation. Integrating the EPR method with satellite imagery facilitates a precise and reliable assessment of shoreline dynamics. The approach necessitates the comparison of at least two shorelines. Initially, a baseline is delineated, approximately parallel to the shoreline trend. Subsequently, vertical transects are spaced at regular intervals along this baseline to demarcate division points. The calculation involves determining the imaging dates for the shoreline positions flanking each transect. The methodology for computation is as follows:

(2) $$\mathrm{EPR}=\frac{d_{2}-d_{1}}{t_{2}-t_{1}}$$

In the formula, d_1_ represents the distance (meters) from the intersection of the vertical line with the earliest shoreline to the baseline; d_2_ is the distance (meters) from the intersection of the vertical line with the latest shoreline to the baseline; t_1_ is the imaging time (years) of the corresponding image for the earliest shoreline; and t_2_ is the imaging time (years) of the corresponding image for the latest shoreline.

## 3. Results and Discussion

### 3.1. Results of Coastline Extraction

**Figure 3:**
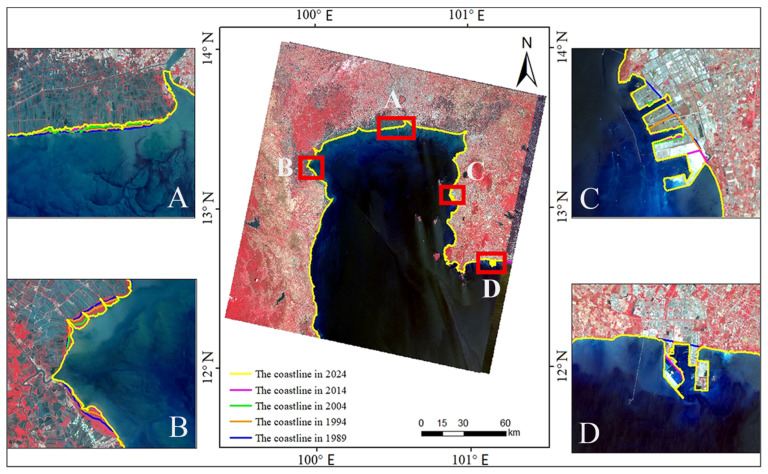
Overlay of the extracted shorelines across five temporal periods (1989, 1994, 2004, 2014, and 2024) in Bangkok Bay. **(A)** identifies shoreline change zones adjacent to Samut Sakhon, Bangkok, and Samut Prakan Provinces, **(B)** identifies shoreline changes at the Klong Yi San Kao Estuary, **(C)** identifies shoreline changes at the Kerry Siam Seaport Terminal, **(D)** identifies shoreline changes at the Map Ta Phut Industrial Port.

Remote sensing images were interpreted using a human–computer interactive method to extract coastline data for five distinct periods. To clarify the temporal variation characteristics of the coastline, a comparative analysis of the five phases of extraction results was conducted. Using the 2024 coastline as the baseline, regions with significant changes (e.g., erosion, accretion, and artificial reclamation) were cropped and magnified to highlight detailed differences (Figure 3).

### 3.2. Analysis of Changes in Coastline Length

**Figure 4:**
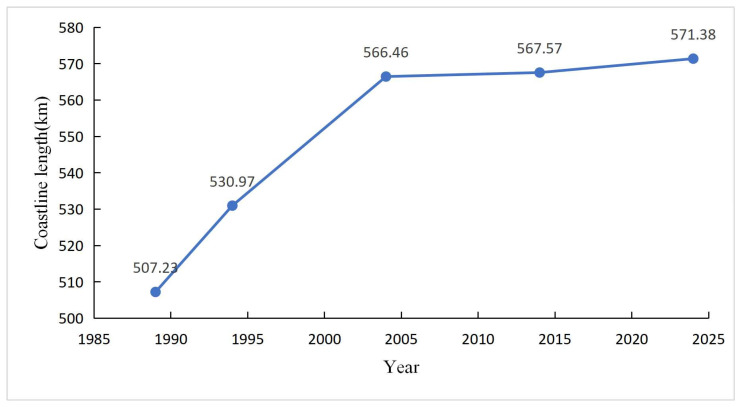
Trend of shoreline length variation in Bangkok Bay, Thailand from 1989 to 2024.

The 35-year growth trajectory of Bangkok Bay’s coastline was characterized by an overall lengthening trend, with the growth rate shifting from an initial rapid phase to a subsequent gradual phase. Specifically, the coastline extended from 507.23 km in 1989 to 571.38 km in 2024 (Figure 4), representing a net extension of 64.15 km over the study period and a cumulative growth rate of 12.65%. Notably, the most significant coastline modification occurred during the 1989–1994 interval, which recorded an average annual change rate of 4.75 m/a. This rate is markedly higher than the mean rate of the entire 35-year period, indicating an intensive period of coastal transformation in the early stage of the study timeframe.

### 3.3. Analysis of Coastline Change Rate

**Figure 5:**
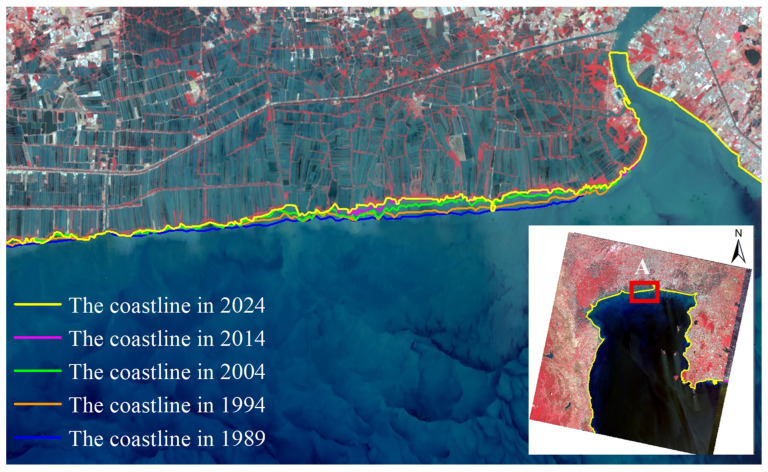
Spatiotemporal evolution of the coastal sections adjacent to Samut Sakhon, Bangkok, and Samut Prakan Provinces (Area A) from 1989 to 2024.

**Figure 6:**
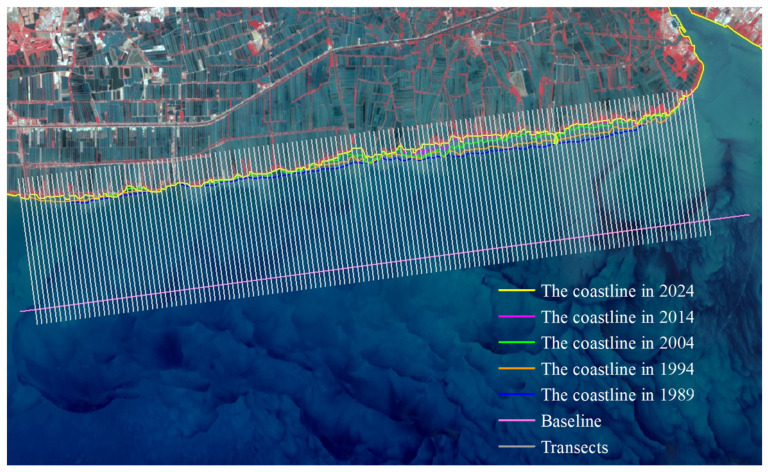
Configuration of multi-temporal shorelines, baseline, and measurement transects for EPR calculation.

**Figure 7:**
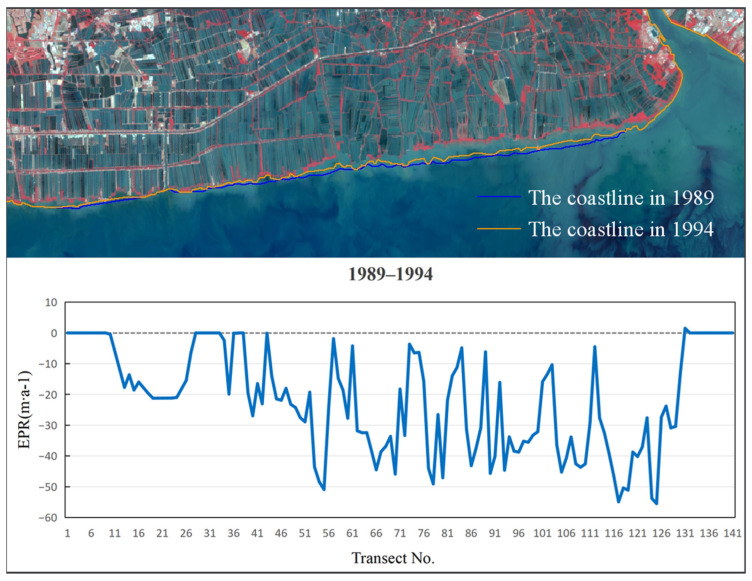
The coastline positions and EPR values from 1989 to 1994.

**Figure 8:**
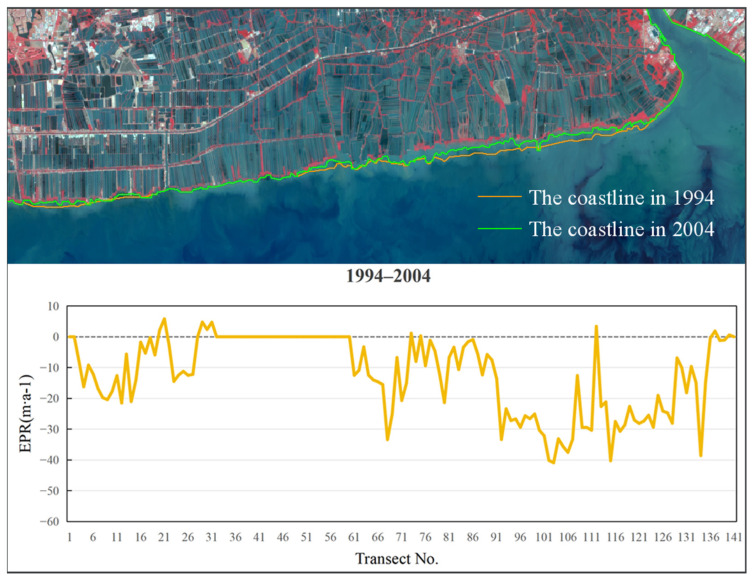
The coastline positions and EPR values from 1994 to 2004.

**Figure 9:**
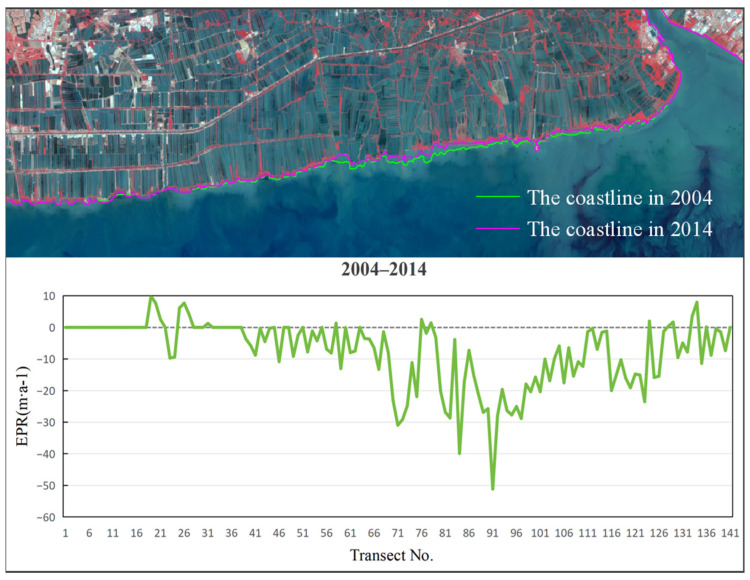
The coastline positions and EPR values from 2004 to 2014.

**Figure 10:**
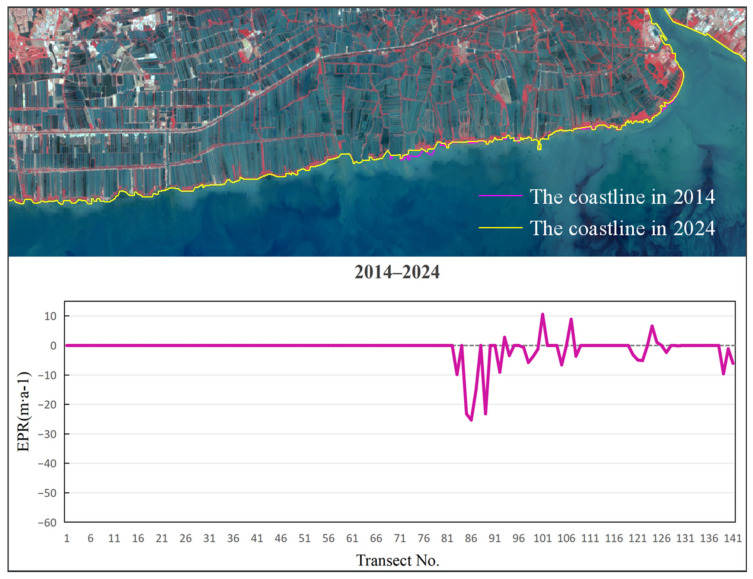
The coastline positions and EPR values from 2014 to 2024.

**Figure 11:**
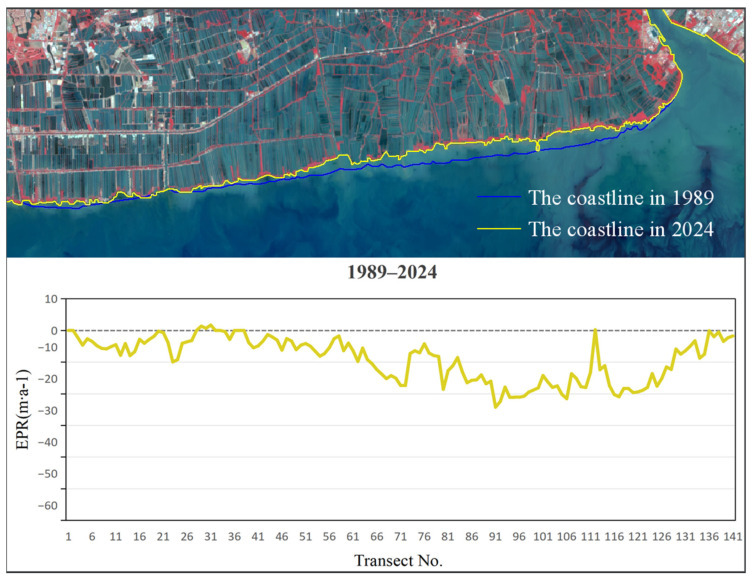
The coastline positions and EPR values from 1989 to 2024.

To analyze the rate of coastline, change in Bangkok Bay from 1989 to 2024, this study selected the coastal sections adjacent to Samut Sakhon, Bangkok, and Samut Prakan Provinces as the representative study segment (Figure 5). Remote sensing images were interpreted using a human–computer interactive method to extract coastline data for five distinct periods. The calculation of End Point Rate (EPR) values across all specified time intervals was conducted using uniformly spaced (200 m) transect lines set normal to the baseline (Figure 6), a method facilitating precise capture of coastline evolution through time and distinct phases. The subsequent figures (Figure 7, Figure 8, Figure 9, Figure 10 and Figure 11) present these EPR results, with the sign convention denoting accretion (positive) and erosion (negative).

Examinations of the EPR between 1989 and 1994, as depicted in Figure 7, revealed a period of net erosion, with an average rate of −21.61 m/a. The maximum erosion was observed near section No. 125, peaking at −55.49 m/a. The highest deposition rate of 1.49 m/a was observed near section No. 131. From 1994 to 2004 (Figure 8), the average EPR decreased to −12.09 m/a, and the maximum erosion rate of −40.92 m/a was identified near section No. 103. The highest deposition rate of 5.82 m/a occurred near section No. 21. During 2004 to 2014 (Figure 9), the average EPR was −7.75 m/a, with the maximum erosion rate of −51.15 m/a near section No. 91, and the highest deposition rate of 9.86 m/a near section No. 19. From 2014 to 2024 (Figure 10), the average EPR further decreased to −0.95 m/a, with the maximum erosion rate of −25.32 m/a near section No. 86. The highest deposition rate of 10.57 m/a was observed near fault plane number 101. Over the entire period from 1989 to 2024 (Figure 11), the average EPR was −9.03 m/a, with the maximum erosion rate of −24.23 m/a near section No. 91 and the highest deposition rate of 1.7 m/a near section No. 31. Detailed data are presented in Table 2. Overall, the coastline of Samut Sakhon–Bangkok–Samut Prakan Provinces experienced net erosion from 1989 to 2024. The most severe erosion occurred during the 1989–1994 period, with the highest erosion rate and the most significant coastal retreat. In contrast, the coastal erosion rate was relatively low during the 2014–2024 period, while the accretion rate was comparatively higher.

The results of this study could serve as key inputs to a broader decision-support framework for coastal zone management. By integrating the spatial patterns of erosion, accretion, and shoreline dynamics identified in this research, planners and policymakers can enhance coastal risk assessments and prioritise intervention areas. Incorporating these findings into optimisation and multi-criteria decision-making tools would enable more data-driven strategies for designing protective infrastructure, managing land use, and strengthening resilience along the Bangkok Bay coastline.

### 3.4. Analysis of Causes of Coastline Change

Long-term analysis of coastline changes in a representative area of Bangkok Bay reveals that the evolutionary dynamics can be attributed to two principal factors: natural processes and human activities.

#### 3.4.1. Natural Factors

Natural factors influencing the coastal zone of Bangkok Bay encompass tidal action, sea-level changes, storm surges, river sediment transport, geological tectonic movements, ocean waves, tidal currents, and biological activities, all of which can impact the location and morphology of the coastline. The predominant natural factors affecting Bangkok Bay are:

- Tidal action: The sea level in Bangkok Bay is primarily influenced by astronomical tides, meteorological winds, and the pressure and discharge of large rivers. The cyclic nature of tides results in the continuous erosion and accretion of coastal areas [29].
- Sea-level change: Coastal erosion is exacerbated by global climate change, predominantly through the mechanism of sea-level rise. Bangkok Bay, located in the northern Gulf of Thailand, experiences distinct wet and dry seasons. Trisirisatayawong et al. documented that the Gulf of Thailand is experiencing sea-level rise at a rate of 3–5.5 mm/year, significantly faster than the global average. Owing to its low topographic relief and extensive flat areas, Bangkok Bay is highly susceptible to threats from sea-level rise and coastal flooding, causing the coastline to recede inland [34].
- Storm surge: Extreme weather events, such as typhoon-induced storm surges, can lead to significant alterations of the coastline.
- Biological effects: The roots of mangroves and other biological structures can stabilize the soil, enhance the stability of the coastal zone and provide a protective effect on the shoreline.

#### 3.4.2. Anthropogenic Factors

Anthropogenic activities significantly impact the coastal zone of Bangkok Bay, including coastal development, aquaculture, groundwater extraction, climate change, river diversion and regulation, and pollutant discharge. These factors influence the location and form of the coastline to varying degrees. The primary anthropogenic factors affecting Bangkok Bay are:

- Coastal development: Urbanization through land reclamation and port construction has led to rapid alterations in coastline types. Expansion seaward has extended the coastline, creating numerous convex features, such as the Laem Chabang Port in Bangkok Bay. Coastal structures like breakwaters and revetments, intended to prevent erosion, may disrupt natural coastal processes [35].
- Aquaculture: Many residents in the coastal areas of Bangkok Bay rely on aquaculture and agriculture as their primary livelihoods. In pursuit of commercial gains, extensive mangrove destruction for shrimp farming has occurred. These activities can modify seabed topography and water quality, pollute the environment, degrade marine water quality, reduce coastal environmental carrying capacity, and indirectly affect the coastline. The loss of mangroves also diminishes soil stability, making the coastline more susceptible to seawater erosion and causing it to retreat inland [36].
- Coastal groundwater extraction: Over-extraction of groundwater can result in land subsidence, affecting the coastline. For instance, the over-extraction of groundwater to meet the high urban water demand in Bangkok, resulting from its large population, has caused the coastal zone to subside. This relative drop in land level accelerates coastal erosion and prompts inland shoreline migration.
- Climate change: While climate change is part of Earth’s natural cycle, it is now accelerating at unprecedented rates due to human activities. This acceleration is widely regarded as an anthropogenic factor, distinctly altering global environmental systems.

Human interventions, including aquaculture-driven mangrove clearance and port expansion, have disrupted natural sediment pathways. Comparable cases were reported in North Sinai and Songkhla, Thailand, where mangrove loss accelerated shoreline degradation [14,25]. Erosion hotspots threaten nearby infrastructure, underscoring the need for proactive buffer zones and ICZM. Analogously, strategic environmental assessment frameworks have been implemented in Spain’s Valencian Coast to evaluate risks to transport and port facilities and guide sustainable infrastructure planning [37]. In conclusion, the synergistic effects arising from the interplay of the identified natural and anthropogenic drivers govern the morphological trajectory of Bangkok Bay’s coastline over extended temporal scales.

### 3.5. Comparative Analysis of Tropical Coastal Zones

The shoreline dynamic characteristics of Bangkok Bay identified in this study are not only highly consistent with the regional patterns of tropical coasts in Latin America and Africa but also align with the universal evolutionary models of global tropical coastal zones, fully verifying the representativeness and universality of the research findings.

From a regional perspective, Bangkok Bay’s shoreline dynamics align closely with those documented in Latin American tropical coasts. Studies conducted in the Amazon and Pará regions of Brazil have demonstrated that reduced sediment supply and mangrove loss have resulted in erosion rates comparable to the intense retreat recorded in Bangkok Bay during 1989–1994 [38,39]. Similarly, coastal segments in Ecuador and Colombia exhibit alternating phases of erosion and stabilization, driven by seasonal currents and land-use pressures [40], which mirrors Bangkok Bay’s trajectory of strong early-stage erosion followed by decelerated change rates post-2014. Comparable shoreline behavior has also been reported along tropical African coasts: in the Niger Delta, mangrove clearing, land subsidence, and coastal development have generated shoreline retreat rates analogous to those quantified in this study [41]; DSAS-based analyses of the Gulf of Guinea, Tanzania, and Mozambique coasts have revealed a pattern of rapid erosion in sediment-limited environments followed by partial long-term stabilization [42,43,44], which closely reflects Bangkok Bay’s transition from high-magnitude erosion to moderate change rates.

From a global perspective, the findings of this study further align with well-established patterns across tropical coastlines worldwide. A key characteristic of sediment-starved deltas globally is the occurrence of peak erosion rates during the initial decades of monitoring [45,46], which is consistent with the severe retreat (−55.49 m/a maximum) documented in Bangkok Bay between 1989 and 1994. The role of mangrove loss in accelerating shoreline recession has been widely corroborated across tropical regions [27,35], matching the erosion hotspots identified near intensive aquaculture and land-clearing areas in Bangkok Bay. Additionally, coastal development—including port expansion and anthropogenic land reclamation—is a globally recognized driver of tropical shoreline modification [25,47], as evidenced by localized shoreline shifts near Bangkok’s industrial zones. Furthermore, the use of DSAS and Landsat imagery in this research is consistent with best practices in tropical coastal monitoring, as similar approaches have been widely validated in Africa, Latin America, and Asia [48,49,50,51], further highlighting the reliability and generalizability of the analytical workflow.

### 3.6. Engineering Implications of Shoreline Change

The results of erosion and accretion in Bangkok Bay are of direct engineering value. Regions where the receding areas have a steady repetition can be more dangerous to the key coastal framework, including roads, ports and industrial regions. Such results justify the necessity of protective buffer zones and pre-planning design techniques, such as the establishment of revetments or seawalls or natural-focused interventions such as mangrove restorations. Additionally, understanding long-term coastal change dynamics (including wave patterns) is essential for developing effective adaptation strategies in vulnerable coastal areas, as highlighted by Vousdoukas et al., who showed that climate change could lead to significant shoreline retreat by the end of the century [52]. The large-scale erosion statistics between 1989 and 1994 (−21.61 m/a) in Bangkok Bay also seems in accord with the patterns observed in other deltaic coastal areas where human modifications, such as embankment construction, have interrupted sedimentation and altered tidal patterns, leading to accelerated land loss, similar mechanisms have been documented in the Ganges-Brahmaputra Delta [45]. However, long-term analyses of Mekong Delta shoreline change highlight the dominant role of reduced sediment supply in enhancing coastal erosion over recent decades, and they show spatial and temporal variations in deposition and erosion patterns along different segments of the delta coast [53]. Shoreline retreat in Bangkok Bay is highly influenced by tidal energy and wave action and is related to sediment dynamics. Similar deltaic coasts in Southeast Asia, including the Mekong Delta, have been exposed to rapid erosion under weak sediment supply and strong tidal forcing [46].

The methodological framework presented in this study using MNDWI-based water extraction and DSAS/EPR shoreline change analysis can also be strengthened further with the addition of modelling or optimization approaches. Linking GIS-based measures with statistical or artificial intelligence-based models (e.g., machine learning regression, fuzzy logic, or neuro-fuzzy systems) would improve predictions of shoreline evolution, while allowing simulation under varying climatic or anthropogenic scenarios.

Additionally, spatial products of shoreline change can be important inputs for optimization models, which can be used for sitting coastal protection structures such as seawalls, breakwaters, and mangrove restoration areas. The combination of erosion and accretion information with cost, ecological, and spatial suitability will allow for optimal configurations to be located that balance engineering efficiency and long-term ecosystem resilience. This combined GIS–optimization platform takes current descriptive analysis and moves it into an actionable decision-support mechanism for sustainable coastal management.

While this study centered on the application of the MNDWI alongside the DSAS/EPR framework for shoreline change analysis, subsequent studies may benefit from making comparisons with additional analytical and forecast modelling approaches. Regression-based approaches such as linear or polynomial trend estimates may statistically support observed shoreline dynamics while data-driven modelling approaches such as an artificial neural network or hybrid neuro-fuzzy systems may capture the non-linear and spatially heterogeneous behavior of erosion- deposition. A comparative analysis of these models and approaches would serve to not only enhance the analytical rigour of shoreline prediction but also indicate the contexts in which each performs best. In addition to the predictive reliability of the models, any consideration of models would extend the methodological innovation of GIS-based coastal research.

## 4. Limitations and Future Directions

This study is limited by its reliance on medium-resolution Landsat imagery, which, although useful for long-term monitoring, may overlook small-scale shoreline changes due to spatial resolution and tidal variation during image capture. Validation was mainly visual, and the lack of extensive ground-truth surveys or UAV-based observations reduces overall accuracy. Future research should integrate higher-resolution satellite datasets, UAV photogrammetry, and field GPS surveys to improve precision. Stochastic modeling could also help address uncertainties in shoreline projections. Combining remote sensing with socio-economic and hydrodynamic models would provide a more comprehensive perspective on human–environment interactions. Extending similar approaches to other coastal provinces in Thailand would further support integrated and resilient coastal management.

The methodological framework designed in this research, which integrates MNDWI-based water extractions with DSAS/EPR shoreline change techniques, could be further enhanced by incorporating modelling or optimization approaches. By combining the GIS-based findings with statistical models (machine learning regression, fuzzy logic, and/or neuro-fuzzy), or incorporating artificial intelligence, the predictions of shoreline dynamics could be improved, and predictions could be simulated under different climactic or anthropogenic conditions.

Additionally, spatial products of shoreline change could be used to inform optimization models to guide the placement of coastal protection structures including seawalls, breakwaters, and mangrove restoration zones. By incorporating both erosion and accretion data with cost, ecological, and spatial-suitability variables, optimization can identify the “best” combinations of options to provide engineering efficiency and ecological resilience. This GIS-optimization framework shifts our current descriptive analysis to a decision-support system that considers opportunity costs related to coastal management actions and sustainability.

## 5. Conclusions and Implications

In this study, the coastline of Bangkok Bay, encompassing eight provinces from Phetchaburi to Rayong, is selected as the research area. Landsat satellite images captured every 5 or 10 years between 1989 and 2024 are utilized to investigate the temporal and spatial variations in the length of the Bangkok Bay coastline. The application of the End Point Rate (EPR) provides a quantitative assessment of change rates for selected representative coastal segments. Additionally, the underlying causes of coastline alterations are analyzed. Subsequent research could focus on integrating the determinants of coastline change within the study area, quantifying the specific contributions of each factor, and evaluating the ecological and environmental consequences of coastline modifications. The primary results and conclusions derived from this research are presented below: the growth trajectory of Bangkok Bay’s coastline over the 35-year period shows an overall lengthening, with the rate of increase gradually slowing over time. In addition, the coastline of Samut Sakhon–Bangkok–Samut Prakan Provinces experienced net erosion. Over the entire period from 1989 to 2024, the average EPR was −9.03 m/a, with the maximum erosion rate of −24.23 m/a and the highest deposition rate of 1.7 m/a. This study’s findings are valuable for coastal risk management and engineering. The documented erosion and accretion rates will contribute to the placement of structural defenses such as seawalls, groynes, and revetments as well as nature-based solutions like the restoration of mangroves.

The findings of this study provide several important implications for science, management, and policy. Firstly, they demonstrate the effectiveness of integrating multi-temporal satellite imagery with DSAS and GIS as a reliable framework for long-term shoreline monitoring in data-scarce coastal environments. This methodological contribution highlights the potential of medium-resolution archives, such as Landsat, for reconstructing decades of coastal dynamics at low cost. Secondly, the results establish a quantitative baseline of erosion and accretion patterns for Bangkok Bay, which can be used as reference data in future hydrodynamic modeling and vulnerability assessments. Thirdly, by documenting spatially explicit erosion hotspots, the study offers evidence to guide targeted interventions, prioritization of high-risk areas, and evaluation of coastal adaptation options. Finally, these insights contribute to the broader discourse on coastal resilience in Southeast Asia, where urban growth, sea-level rise, and ecosystem degradation increasingly intersect with shoreline change.

## Figures and Tables

**Table 1:** Utilization of Multi-Temporal Landsat Satellite Data for Coastal Change Detection.

No.	Year	Title 3	Spectral Resolution (Meter)
1	1989	Landsat-5	30 m
2	1994	Landsat-5	30 m
3	2004	Landsat-5	30 m
4	2014	Landsat-8	30 m
5	2024	Landsat-8	30 m

Landsat data from the USGS website (https://earthexplorer.usgs.gov/, accessed on 20 August 2024).

**Table 2:** EPR Values Across Different Temporal Intervals.

No.	Temporal Interval	Mean Erosion Rate (m/a)	Maximum Erosion Rate (m/a)	Maximum Deposition Rate (m/a)
1	1989–1994	−21.61	−55.49	1.49
2	1994–2004	−12.09	−40.92	5.82
3	2004–2014	−7.75	−51.15	9.86
4	2014–2024	−0.95	−25.32	10.57
5	1989–2024	−9.03	−24.23	1.7

## Data Availability

The original contributions presented in this study are included in the article. Further inquiries can be directed to the corresponding author.

## Abbreviations

The following abbreviations are used in this manuscript:

DSAS	Digital Shoreline Analysis System
EPR	End Point Rate
GIS	Geographic Information System
ICZM	Integrated Coastal Zone Management
MNDWI	Modified Normalized Difference Water Index
RS	Remote Sensing
USGS	United States Geological Survey
